# Supplementary material for: Activating cavity by electrons
Source: Commun Phys. 2023 May 22;6(1):111. doi: 10.1038/s42005-023-01227-8 (PMC11041782; doi:10.1038/s42005-023-01227-8)
Supplement: Supplementary file 1 — Supplementary Information [file 42005_2023_1227_MOESM1_ESM.pdf]

Activating Cavity by Electrons
Supplementary Information

Lorenz S. Cederbaum[1*] and Jacqueline Fedyk[1]
[1] *Theoretische Chemie, Physikalisch-Chemisches Institut, Universität Heidelberg, Im Neuenheimer Feld 229, Heidelberg D-69120, Germany*

(Dated: April 26, 2023)

SUPPLEMENTARY CONTENTS

Supplementary Note 1: Resonance energies
Supplementary Note 2: Results for resonances close to threshold
Supplementary Note 3: Impact of cavity photon lifetime
Supplementary Note 4: A brief remark on more systems

* Lorenz.Cederbaum@pci.uni-heidelberg.de

SUPPLEMENTARY NOTE 1: RESONANCE ENERGIES

The structure of the T-Matrix, Eq. (15) is that of a discrete electronic state of energy ϵ_c embedded in the continuum and interacting with it [1]. In a cavity, the discrete state's energy is explicitly $\epsilon_c = E_0^{N+1} - E_0^N + \hbar\omega_c$ and describes the energy of the hybrid anion-light state $|\Phi_0^{N+1}, 1_c\rangle$ relative to the energy of the target. The energy of the discrete state is shifted by $\Delta(\epsilon_p)$ and the discrete state acquires a finite lifetime by $\Gamma(\epsilon_p)$ via its interaction with the continuum. It is common to define the resonance energy as the solution of [1]

$$E_{res} = \epsilon_c + \Delta(E_{res}). \tag{1}$$

I.e., where for $\epsilon_p = E_{res}$ the real part of the denominator of the T-matrix vanishes. Assuming that the imaginary part $\Gamma(\epsilon_p)/2$ varies smoothly with energy, the scattering cross section peaks at the resonance energy $\epsilon_p = E_{res}$.

While the width function $\Gamma(\epsilon_p)$ vanishes for negative argument, the shift function $\Delta(\epsilon_p)$ does not, see Eq. (23). To have a complete picture of the situation of the resonance energy resulting from Eq. (1), we depict the shift function in Fig. S1 also for negative energy values. We stress that solutions of Eq. (1) at negative energies are meaningful and correspond to bound states. The coupling with the continuum of a discrete state lying in the continuum can give rise to a bound state below the threshold of the continuum if this coupling is sufficiently strong. Since the shift function $\Delta(\epsilon_p)$ depends quadratically on the cavity coupling strength g_0, one can control the strength of the coupling and thus the appearance of the structure of the cross section and capture probability, see also section .

The solutions of Eq. (1) for the scattering cross sections addressed in Fig. 3 of the main text are depicted in Figs. S2 and S3. Each of these figures is for a given value of $\hbar\omega_c$ and shows three panels related to the different cavity coupling values of g_0 treated in the main text. Shown are the curves $E = \epsilon_p$ and $E = \epsilon_c + \Delta(\epsilon_p)$, the crossing points of which are solutions of Eq. (1).

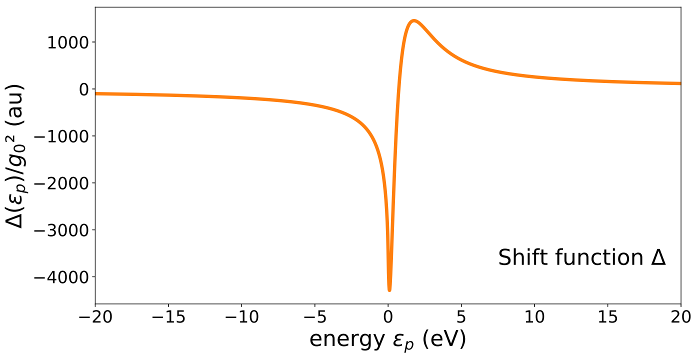

FIG. S1. The entire level shift function Δ. Note that while the width function Γ vanishes for negative argument, this is not the case for Δ. As a consequence, solutions of equation (1) at negative energies are possible and give rise to bound states below the threshold of the continuum.

For the weak coupling case, the energy area around the crossing is smooth and one can anticipate a peaked structure of the cross sections at the energy of the resonance, symmetric for $\hbar\omega_c = 2.5$ eV and less symmetric for $\hbar\omega_c = 1.5$ eV due to the wiggle $\Delta(\epsilon_p)$ makes at low energy. For the other coupling strengths the situation is more interesting. For $\hbar\omega_c = 2.5$ eV, the resonance energy is rather high and a peak can be seen in the cross section close to this energy, and due to the now more pronounced wiggle of $\Delta(\epsilon_p)$ and remembering that $\Gamma(\epsilon_p)$ is larger at lower energies, a second peak emerges in the cross sections at low energy (see Fig. 3 of the main text). This lower lying peak is relatively more dominant for the strongest coupling strength, because the energy difference there between the two curves in the lower panel of Fig. S3 is clearly smaller than that in the middle panel. See Eq. (24).

A rather unusual situation occurs for the stronger couplings at $\hbar\omega_c = 1.5$ eV. One can see in the middle panel of Fig. S2 that the two curves $E = \epsilon_p$ and $E = \epsilon_c + \Delta(\epsilon_p)$ are very close to each other from about threshold to their crossing point. This explains the rather spread out structure of the cross section avoiding a two peak structure like that seen for $\hbar\omega_c = 2.5$ eV in Fig. 3 of the main text and discussed above. The situation is similar for the strongest studied coupling. Here, however, as seen in the lowest panel of Fig. S2, the two curves are not just close to each other over an extended energy range, but even exhibit more than a single crossing point. We may conclude that the cross sections, as well as the resonances can change substantially by varying the parameters of the quantum light used.

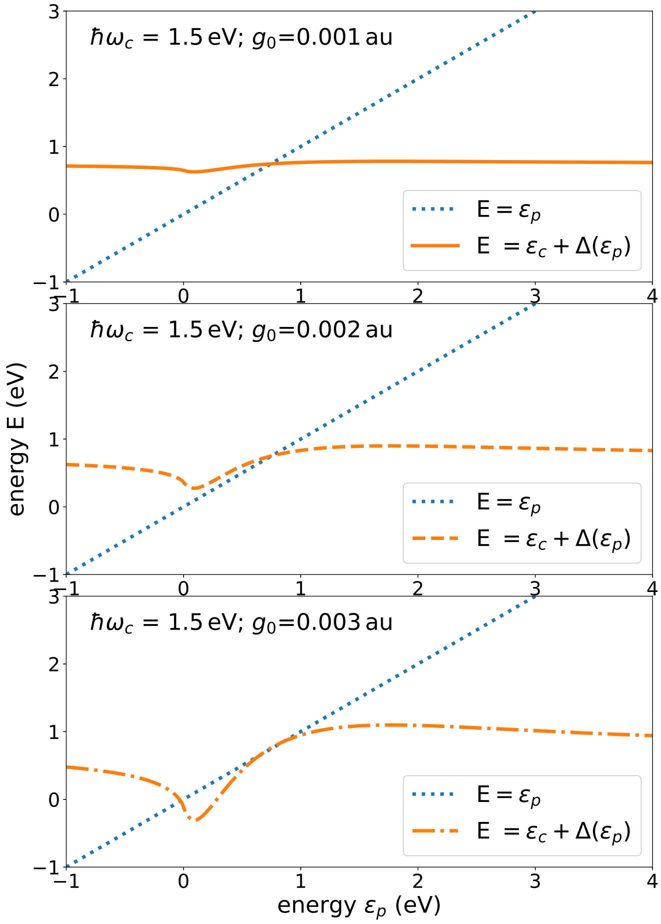

FIG. S2. On the energy of the resonance for the H atom and $\hbar\omega_c = 1.5$ eV. Shown are the two curves $E = \epsilon_p$ and $E = \epsilon_c + \Delta(\epsilon_p)$ as a function of energy ϵ_p for three values of the cavity coupling strength g_0. At their crossing point(s), the equation (1) defining the resonance energy is fulfilled. See the text explaining the appearance of the respective scattering cross sections in the light of these curves.

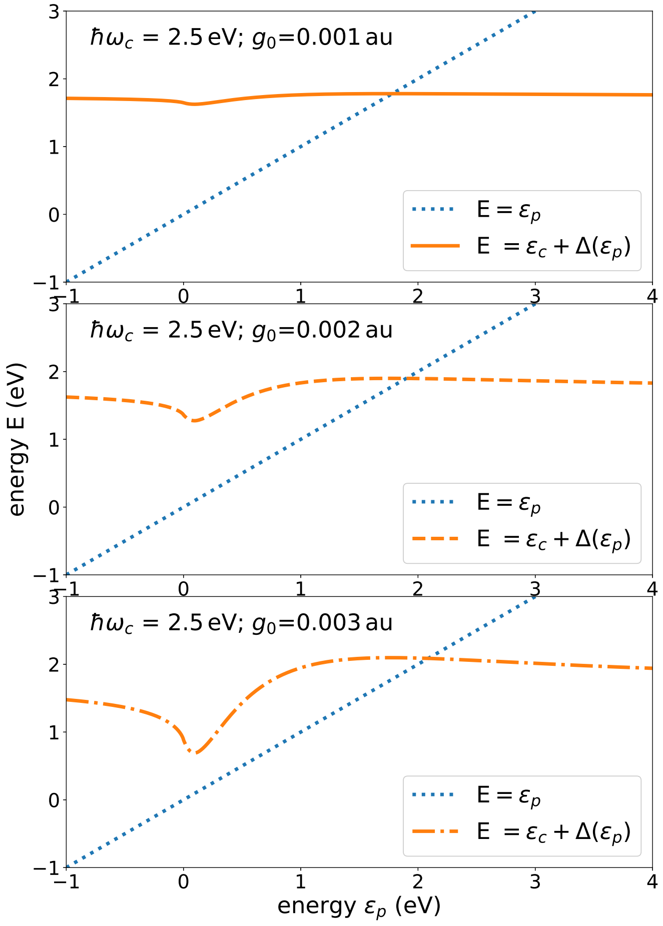

FIG. S3. On the energy of the resonance for the H atom and $\hbar\omega_c = 2.5$ eV. Shown are the two curves $E = \epsilon_p$ and $E = \epsilon_c + \Delta(\epsilon_p)$ as a function of energy ϵ_p for three values of the cavity coupling strength g_0. At their crossing point(s), the equation (1) defining the resonance energy is fulfilled. See the text explaining the appearance of the respective scattering cross sections in the light of these curves.

SUPPLEMENTARY NOTE 2: RESULTS FOR RESONANCES CLOSE TO THRESHOLD

In general, one can expect strong effects on the cross section if the resonance is close to threshold [1]. Whether a resonance of a free atom or molecule is close to threshold, is dictated by nature. In cavity, one can, in principle, tune the cavity such that the resonance gets close to threshold. Indeed, as the shift function varies substantially close to threshold, see Fig. S1, one may anticipate strong threshold effects as demonstrated in the main text.

Let us return to the example of the H atom and choose the cavity frequency such that for a given coupling strength the position of the emerging resonance is close to threshold. The calculated cross sections are collected in Fig. 5 of the main text for three values of the coupling strength. A resonance close to threshold has been shown there to lead to a high and peaked cross section close to threshold. In the upper panel, the photon energy $\hbar\omega_c = 0.9$ eV creates a light-induced resonance close to threshold for the weakest coupling strength. As seen in the figure, the respective cross section strongly dominates those for the stronger coupling values. To better understand the finding that the weak coupling peak strongly dominates those for the stronger couplings, we compute the resonance positions for $\hbar\omega_c = 0.9$ eV and all the three coupling strengths employed. Similarly as done above in Figs. S2 and S3 for other values of $\hbar\omega_c$, we plot the two curves the two curves $E = \epsilon_p$ and $E = \epsilon_c + \Delta(\epsilon_p)$ and look for their crossing points which define the resonance energy position. The results for all three values of the cavity coupling strength are drawn in Fig. S4 for $\hbar\omega_c = 0.9$ eV. It is seen in the upper panel that as expected, the resonance for the weakest coupling is close to threshold. Because the shift function Δ grows quadratically with the coupling strength, the respective solutions the equation $E_{res} = \epsilon_c + \Delta(E_{res})$ drop below the threshold and instead of a resonance, a bound hybrid state appears below threshold. The impact of these bound states on the respective cross sections is much weaker than that of a resonance close to threshold in spite of the larger coupling strengths.

The other panels of Fig. 5 in the main text demonstrate that one can selectively enhance the cross section for a desired coupling strength. For any value of g_0, there is a value $\hbar\omega_c$ which creates a resonance close to threshold. At the same $\hbar\omega_c$, the resonance is pushed below the threshold and becomes a bound state for larger values of g_0, and pushed away from threshold and has thus less impact on the cross section for smaller values of g_0.

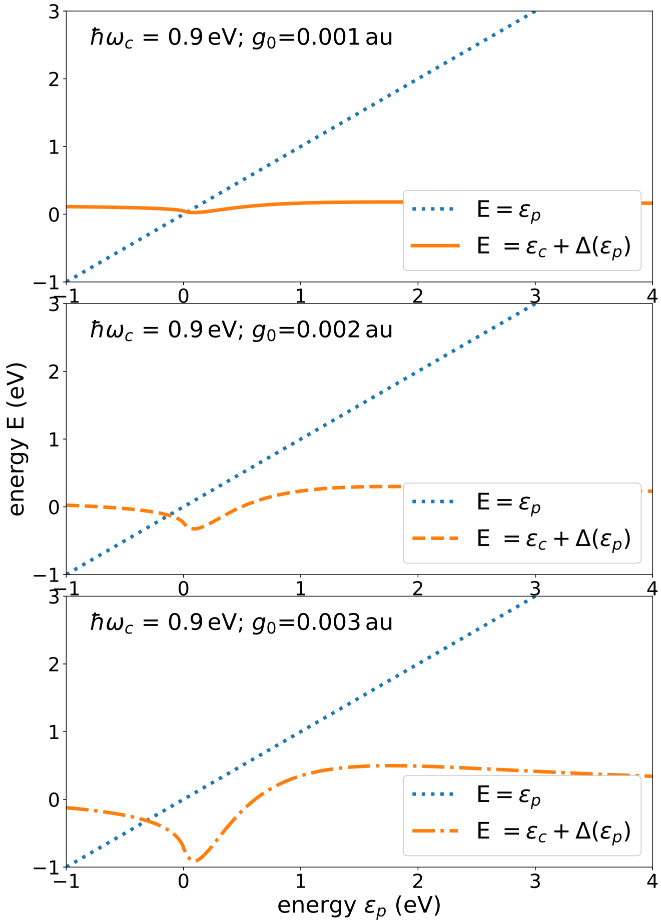

FIG. S4. On the energy of the resonance for the H atom and $\hbar\omega_c = 0.9$ eV. Shown are the two curves $E = \epsilon_p$ and $E = \epsilon_c + \Delta(\epsilon_p)$ as a function of energy ϵ_p for three values of the cavity coupling strength g_0. At their crossing point, the equation (1) defining the resonance energy is fulfilled. For $\hbar\omega_c = 0.9$ eV, the hybrid resonance for the smallest cavity coupling strength is close to threshold and for the stronger couplings it is pushed below threshold and becomes a hybrid bound state.

Since nowadays cavities are lossy and the cavity photon has a finite lifetime, we have redone the calculations of the cross section with $\Gamma_{phot} = 33$ meV, i.e., the lifetime of the cavity has been assumed to be 20 fs. The results are collected in Fig. S5. It is seen that as found for the situation without cavity losses, the cross section of the resonance near threshold strongly dominates the cross sections for the other coupling strengths at the same cavity frequency. However, comparing with the results without losses shown in Fig. 5 of the main text, one finds that there is a strong impact of the cavity lifetime on the cross sections of the resonances close to threshold! This is of particular interest. For many targets the electron affinity can be rather small compared to that of the H atom. Being close to threshold implies that one can use low frequency cavities for which there are cavities of longer lifetime [2]. The measured cross sections reflect sensitively the cavity photon lifetime for resonances at threshold.

To complement the discussion of the cross section of resonances close to threshold, we briefly discuss also the capture probability of the same resonances. The respective data is shown in Fig. S6 for the same conditions as for the cross sections shown in Fig. S5.

For completeness we would like to show the impact of the finite lifetime on the cross sections for resonances far from threshold depicted in Fig. 3 of the main text for the situation without losses, and, on the capture probability depicted in Fig. 4 of the main text with losses. The cross sections computed including the losses can be seen in Fig. S7. Comparing with Fig. 3 of the main text shows some small impact of the losses, very negligible compared to the impact the losses have for resonances close threshold. The capture probabilities computed without taking losses into account are collected in Fig. S8.

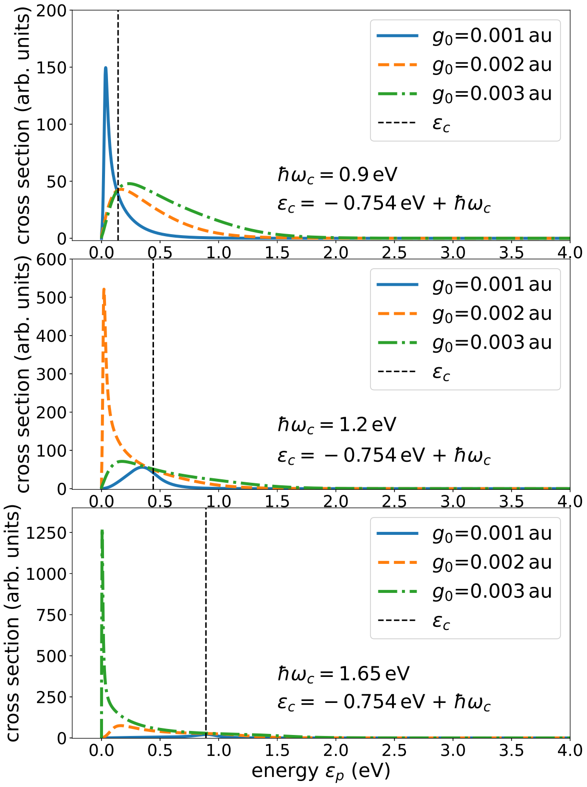

FIG. S5. Illustrative examples of cross sections peaking close to threshold by controlling the cavity frequency for different coupling strengths. In contrast to the ideal cavity in the respective figure (5) of the main text, the present cavity is lossy. The lifetime of the cavity has been assumed to be 20 fs, i.e., $\Gamma_{phot} = 33$ meV. The energetic position of the discrete state embedded in and interacting with the continuum is drawn as a broken vertical line. Its energy depends linearly on the cavity frequency. This discrete state is shifted downwards in energy due to its interaction with the continuum and becomes a resonance due to its coupling to the continuum. As the shift depends quadratically on the coupling strength g_0, one can by choosing the photon energy $\hbar\omega_c$ control the location of the resonance. A resonance close to threshold leads to a high and peaked cross section. The photon energy $\hbar\omega_c = 0.9$ eV brings the resonance for $g_0 = 0.001$ au close to threshold where it dominates the cross sections of the larger coupling strengths. In analogy, the other values of the photon energy shown are chosen to bring the resonance close to threshold for the other values of the coupling strength. Comparison with Fig. 5 of the main text demonstrates that the measured cross sections reflect sensitively the cavity photon lifetime for resonances at threshold.

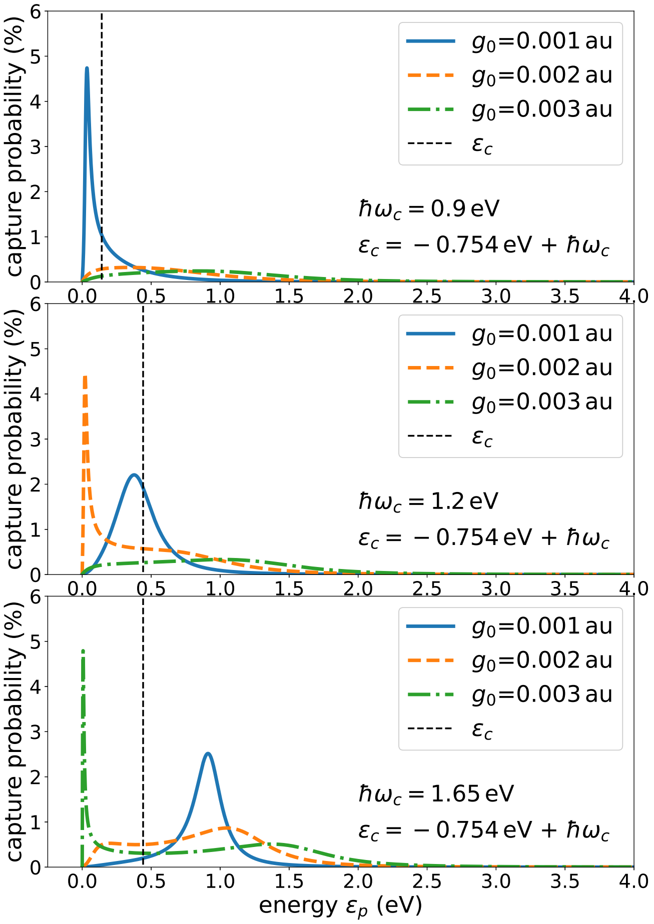

FIG. S6. Capture probability for resonance close to threshold. Shown are the capture probabilities corresponding to the cross sections depicted in Fig. S5.

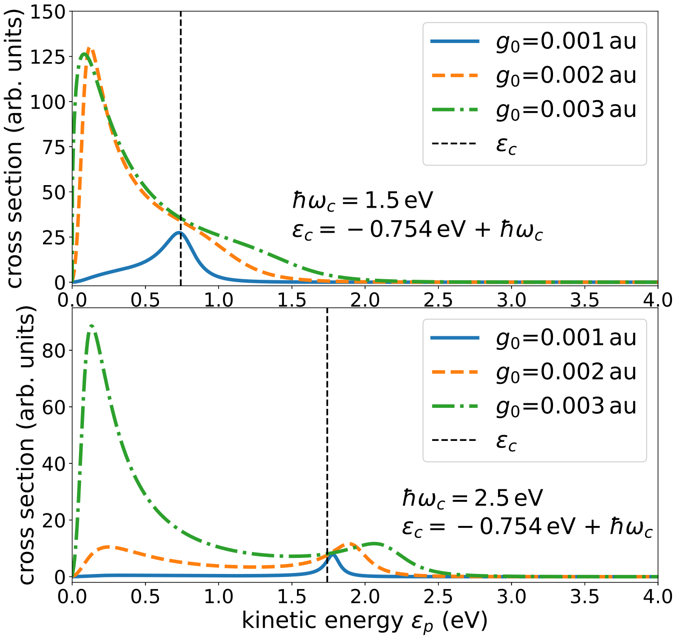

FIG. S7. Illustrative examples of the electron scattering cross section for H atom in a cavity. Shown are cross sections for several values of the coupling strength g_0 and photon energy $\hbar\omega_c$ of the cavity. The energetic position of the discrete state embedded in and interacting with the continuum is drawn as a broken vertical line. Cavity losses (lifetime of the cavity of 20 fs, i.e., $\Gamma_{photon} = 33$ meV) have been taken into account. The results are to be compared with those shown in Fig. 3 of the main text, where cavity losses have not been considered.

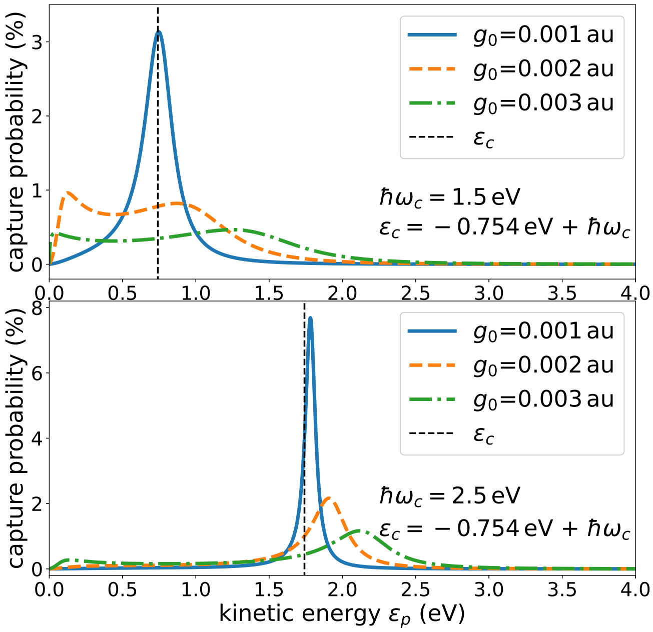

FIG. S8. Illustrative examples of the electron capture probability by the H atom in a cavity. Shown are capture probabilities for several values of the coupling strength g_0 and photon energy $\hbar\omega_c$ of the cavity. No cavity losses are included. The results are to be compared with those shown in Fig. 4 of the main text, where cavity losses are taken into account leading to a lifetime of the cavity of 20 fs, i.e., $\Gamma_{photon} = 33$ meV. The probability relates to electrons in a kinetic energy interval of $\delta_{\epsilon_p} = 0.01$ eV. The energetic position of the discrete state embedded in and interacting with the continuum is drawn as a broken vertical line.

SUPPLEMENTARY NOTE 3: IMPACT OF CAVITY PHOTON LIFETIME

Today, a large variety of cavities exist. The most common types of cavities can be divided into two main groups - optical and plasmonic cavities [3], wherein the first ones the matter-light interaction is based on collective effects, and in the latter ones strong coupling can be even achieved with only one molecule. Optical cavities are often realized as Fabry-Pérot cavities by planar mirrors. Depending on the materials of these mirrors the properties of the cavities differ drastically resulting in lifetimes of high-Q Fabry-Pérot cavities on the order of picoseconds and microcavities on the order of several tens of femtoseconds. In comparison, plasmonic nanocavities are even lossier with typical lifetimes under 10 fs.

It is interesting to see how our results depend on the photon lifetime. We have, therefore, additionally computed capture probabilities and cross sections for lifetimes in the characteristic ranges for high-Q Fabry-Pérot cavities (5 ps) [4–6] and plasmonic nanocavities (5 fs) [7–11]. A few of the computed results are depicted in Figs. S9 and S10 for the capture probabilities and Figs. S11 and S12 for the cross sections and can be compared with the respective ones shown in Fig. 4 and Fig. S6 for 20 fs lifetime of a microcavity. The coupling constants employed here are consistent with those reported for optical microcavities and plasmonic nanocavities [7–9, 12]. Typically, one finds that the capture probabilities, as well as the cross sections, grow when the lifetime increases.

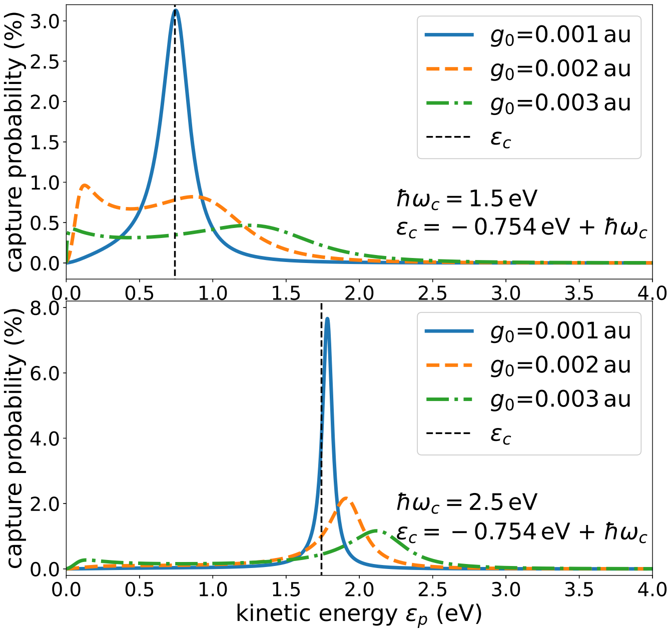

FIG. S9. Illustrative examples of the electron capture probability by the H atom in a cavity. Shown are capture probabilities for several values of the coupling strength g_0 and photon energy $\hbar\omega_c$ of the cavity. The lifetime of the cavity has been assumed to be 5 ps characteristic for high-Q Fabry-Pérot cavities, i.e., $\Gamma_{photon} = 0.13$ meV, and the probability relates to electrons in a kinetic energy interval of $\delta_{\epsilon_p} = 0.01$ eV. The capture probability for 20 fs lifetime of a microcavity is depicted in Fig. 4 of the main text. The energetic position of the discrete state embedded in and interacting with the continuum is drawn as a broken vertical line.

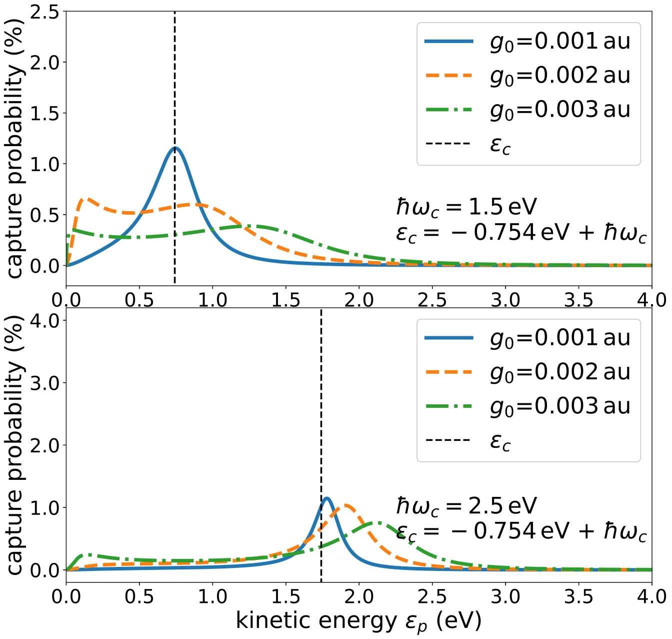

FIG. S10. Illustrative examples of the electron capture probability by the H atom in a cavity. Shown are capture probabilities for several values of the coupling strength g_0 and photon energy $\hbar\omega_c$ of the cavity. The lifetime of the cavity has been assumed to be 5 fs characteristic for plasmonic nanocavities, i.e., $\Gamma_{photon} = 132$ meV, and the probability relates to electrons in a kinetic energy interval of $\delta_{\epsilon_p} = 0.01$ eV. The capture probability for 20 fs lifetime of a microcavity is depicted in Fig. 4 of the main text. The energetic position of the discrete state embedded in and interacting with the continuum is drawn as a broken vertical line.

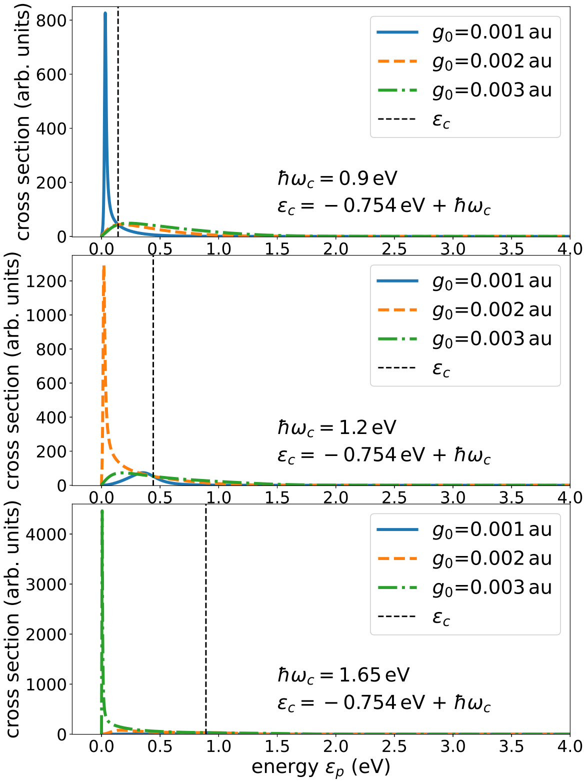

FIG. S11. Illustrative examples of cross sections peaking close to threshold by controlling the cavity frequency for different coupling strengths. In contrast to the cross sections in Fig. S6 where the photon lifetime is 20 fs, the lifetime has been assumed to be 5 ps characteristic for high-Q Fabry-Pérot cavities, i.e., $\Gamma_{phot} = 0.13$ meV. The energetic position of the discrete state embedded in and interacting with the continuum is drawn as a broken vertical line.

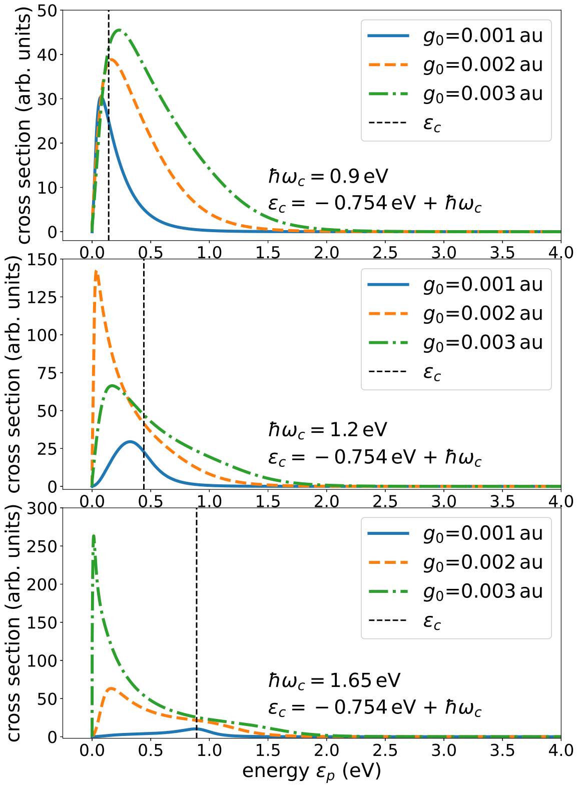

FIG. S12. Illustrative examples of cross sections peaking close to threshold by controlling the cavity frequency for different coupling strengths. In contrast to the cross sections in Fig. S6 where the photon lifetime is 20 fs, the lifetime has been assumed to be 5 fs characteristic for plasmonic nonocavities, i.e., $\Gamma_{phot} = 132$ meV. The energetic position of the discrete state embedded in and interacting with the continuum is drawn as a broken vertical line.

SUPPLEMENTARY NOTE 4: A BRIEF REMARK ON MORE SYSTEMS

Above and in the main text, the scattering of an electron from a single atom or molecule in the cavity has been discussed in some detail. Here, we would like to just touch upon the question of what is to be expected if more systems are available.

Polaritons are commonly discussed in the literature. See also the Introduction and references in the main text. Typically, if we have an ensemble of N identical systems, the normalized superposition $|\Psi_S\rangle = \frac{1}{\sqrt{N}}[|\Phi^N_{1_1}\Phi^N_{0_2}\Phi^N_{0_3}..., 0_c\rangle + |\Phi^N_{0_1}\Phi^N_{1_2}\Phi^N_{0_3}..., 0_c\rangle + ... + |\Phi^N_{0_1}\Phi^N_{0_2}...\Phi^N_{1_N}, 0_c\rangle]$, where $\Phi^N_{0_n}$ are the ground states and $\Phi^N_{1_n}$ excited states of the individual systems, $n = 1, 2, ..., N$, resonantly interacts with the state $|\Psi_0\rangle = |\Phi^N_{0_1}\Phi^N_{0_2}...\Phi^N_{0_N}, 1_c\rangle$ having one cavity photon, giving rise to two plaritons $\frac{1}{\sqrt{2}}[|\Psi_0\rangle \pm |\Psi_S\rangle]$. The other, antisymmetric superpositions, like $|\Psi_{AS}\rangle = \frac{1}{\sqrt{N}}[|\Phi^N_{1_1}\Phi^N_{0_2}\Phi^N_{0_3}..., 0_c\rangle - |\Phi^N_{0_1}\Phi^N_{1_2}\Phi^N_{0_3}..., 0_c\rangle + ...(-1)^{N-1}|\Phi^N_{0_1}\Phi^N_{0_2}...\Phi^N_{1_N}, 0_c\rangle]$ do not couple to the cavity and are called dark states.

In the scenario discussed in the present work, we have one more electron and, in the case of one system, the discrete state $|\Phi^{N+1}_0, 1_c\rangle$ interacts with the continuum $|\Phi^{N+1}(k), 0_c\rangle$ and turns into a resonance. We remind that such resonances exist only due to the presence of the cavity. Now, in analogy to the common situation above, if more identical systems are present in the cavity, we encounter superpositions of states. A symmetric supersposition $|\Psi^{+1}_S\rangle = \frac{1}{\sqrt{N}}[|\Phi^{N+1}_{0_1}\Phi^N_{0_2}\Phi^N_{0_3}..., 1_c\rangle + |\Phi^N_{0_1}\Phi^{N+1}_{1_2}\Phi^N_{0_3}..., 1_c\rangle + ... + |\Phi^N_{0_1}\Phi^N_{0_2}...\Phi^{N+1}_{1_N}, 1_c\rangle]$ and antisymmetric ones, like $|\Psi^{+1}_{AS}\rangle = \frac{1}{\sqrt{N}}[|\Phi^{N+1}_{0_1}\Phi^N_{0_2}\Phi^N_{0_3}..., 1_c\rangle - |\Phi^N_{0_1}\Phi^{N+1}_{1_2}\Phi^N_{0_3}..., 1_c\rangle + ...(-1)^{N-1}|\Phi^N_{0_1}\Phi^N_{0_2}...\Phi^{N+1}_{1_N}, 1_c\rangle]$.

These discrete superposition states couple to the continuum states $|\Psi^{+1}_k\rangle = |\Phi^N_{0_1}\Phi^N_{0_2}...\Phi\Phi^N_{0_N}, k, 0_c\rangle$, where k stands for an electron of momentum k, and turn into resonances, as we found for a single system. The coupling is generated by the cavity and reads

$$\gamma_{Sk} = \langle\Psi^{+1}_k|H_{mc}|\Psi^{+1}_S\rangle = \frac{1}{\sqrt{N}}\sum_n^N \gamma_{k0_n},$$

$$\gamma_{ASk} = \langle\Psi^{+1}_k|H_{mc}|\Psi^{+1}_{AS}\rangle = \frac{1}{\sqrt{N}}\sum_n^N (-1)^{n-1}\gamma_{k0_n}, \tag{2}$$

where γ_{k0_n} is the interaction matrix element of a single system, given in Eq. (5). One sees that the overall coupling can vary strongly as the individual matrix elements γ_{k0_n} can be different from each other as the electron may carry a phase which depends on the position in space. If all γ_{k0_n} are equal, then the overall coupling of the symmetric superposition is

particularly large $\gamma_{Sk} = \sqrt{N}\gamma_{k0}$ and vanishes for the antisymmetric ones. Such a situation may occur for very slow electrons close to threshold, where their de Broglie wavelength is large and may embrace several or many systems. Otherwise, we expect the overall coupling to strongly vary for both the symmetric and all the $N-1$ antisymmetric superposition states. Depending on the size of the various coupling elements γ_{Sk} and γ_{ASk}, the respective resonances will have different energies and widths.

It would be of interest to derive a scattering theory for superposition states. From the above one may expect interesting phenomena for the scattering of electrons from an ensemble of systems in a cavity.

SUPPLEMENTARY REFERENCES

---

[1] Domcke, W. Theory of resonance and threshold effects in electron-molecule collisions: The projection-operator approach. *Phys. Rep.* **208**, 97–188 (1991).

[2] Kockum, A. F., Miranowicz, A., De Liberato, S., Savasta, S. & Nori, F. Ultrastrong coupling between light and matter. *Nat. Rev. Phys.* **1**, 19–40 (2019).

[3] Fregoni, J., Garcia-Vidal, F. J. & Feist, J. Theoretical challenges in polaritonic chemistry. *ACS Photonics* **9**, 1096–1107 (2022).

[4] Velha, P. *et al.* Ultra-high q/v fabry-perot microcavity on soi substrate. *Opt. Express* **15**, 16090–16096 (2007).

[5] Akahane, Y., Asano, T., Song, B.-S. & Noda, S. High-q photonic nanocavity in a two-dimensional photonic crystal. *Nature* **425**, 944–947 (2003).

[6] Foresi, J. S. *et al.* Photonic-bandgap microcavities in optical waveguides. *Nature* **390**, 143–145 (1997).

[7] Benz, F. *et al.* Single-molecule optomechanics in "picocavities". *Science* **354**, 726–729 (2016).

[8] Zengin, G. *et al.* Realizing strong light-matter interactions between single-nanoparticle plasmons and molecular excitons at ambient conditions. *Phys. Rev. Lett.* **114**, 157401 (2015).

[9] Chikkaraddy, R. *et al.* Single-molecule strong coupling at room temperature in plasmonic nanocavities. *Nature* **535**, 127–130 (2016).

[10] May, M. A. *et al.* Nano-cavity qed with tunable nano-tip interaction. *Advanced Quantum Technologies* **3**, 1900087 (2022).

[11] Chen, X. *et al.* Mode modification of plasmonic gap resonances induced by strong coupling with molecular excitons. *Nano Letters* **17**, 3246–3251 (2017).

[12] Liu, B., Menon, V. M. & Sfeir, M. Y. The role of long-lived excitons in the dynamics of strongly coupled molecular polaritons. *ACS Photonics* **7**, 2292–2301 (2020).
